# Combining enabling formulation strategies to generate supersaturated solutions of delamanid: *In situ* salt formation during amorphous solid dispersion fabrication for more robust release profiles

**DOI:** 10.1016/j.ejpb.2022.04.002

**Published:** 2022-05

**Authors:** Tu Van Duong, Hanh Thuy Nguyen, Lynne S. Taylor

**Affiliations:** Department of Industrial and Physical Pharmacy, College of Pharmacy, Purdue University, West Lafayette, IN 47907, United States

**Keywords:** Solid dispersions, Salt formation, Amorphous, Delamanid, Sulfonates, Supersaturation, Stability, Release, ASD, amorphous solid dispersion, DCM, dichloromethane, DL, drug loading, DLM, delamanid, DLS, dynamic light scattering, DMSO, dimethyl sulfoxide, DSC, differential scanning calorimetry, Eud-L, Eudragit L-100, HPLC, high performance liquid chromatography, HPMC, hydroxypropyl methylcellulose, HPMCAS, hydroxypropyl methylcellulose acetate succinate, HPMCP, hydroxypropyl methylcellulose phthalate, IR, infrared, MCC, microcrystalline cellulose, MeOH, methanol, NMR, nuclear magnetic resonance, PBS, phosphate buffer solution, PVPVA, polyvinylpyrrolidone/vinyl acetate, PXRD, powder X-ray diffraction, RH, relative humidity, UV, ultraviolet

## Abstract

Poor solubility is a major challenge that can limit the oral bioavailability of many drugs, including delamanid, a weakly basic nitro-dihydro-imidazooxazole derivative used to treat tuberculosis. Amorphous solid dispersion (ASD) can improve the bioavailability of poorly water-soluble compounds, yet drug crystallization is a potential failure mechanism, particularly as the drug loading increases. The goal of the current study was two-fold: to enhance the stability of amorphous delamanid against crystallization and to improve drug release by developing ASDs containing the salt form of the drug. Various sulfonate salts of delamanid were prepared in amorphous form and evaluated for their tendency to crystallize and undergo chemical degradation following storage at 40 °C/75% relative humidity. Drug release was evaluated by a two-stage dissolution test consisting of an initial low pH stage, followed by transfer to a higher pH medium. For ASDs of the free base, small amounts of crystallinity during preparation were found to limit the drug release. Delamanid salts with sulfonic acids showed considerably improved amorphous stability. Tosylate, besylate, edisylate, and mesylate salts had high glass transition temperatures as well as good chemical and physical stability. In addition, a remarkable improvement in the drug release was observed when ASDs were prepared with these salts in comparison to the free base form. Specifically, ASDs with hydroxypropyl methylcellulose phthalate (HPMCP) at 25% drug loading exhibited near-complete drug release for all four sulfonate salts. These findings suggest that the dual strategy combining salt formation with ASD formation is a promising approach to alter the crystallization tendency and to improve drug release of problematic poorly water-soluble compounds.

## Graphical abstract

**Figure:**
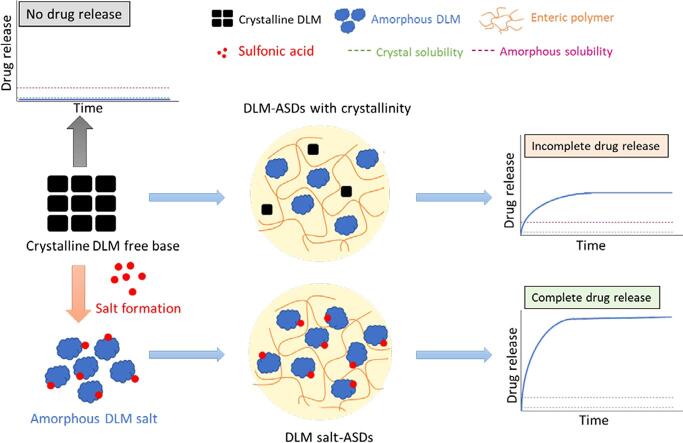

## 1 Introduction

Delamanid (DLM), a nitro-dihydro-imidazooxazole derivative, is indicated for the treatment of multi-drug resistant tuberculosis [1] and is marketed under the brand name Deltyba^TM^. The commercial product is formulated as an amorphous solid dispersion with the enteric polymer, hydroxypropyl methylcellulose phthalate (HPMCP). DLM is a weakly basic compound with a reported pKa of 4.3 [2], and hence exhibits pH-dependent solubility like other weak bases [3], [4]. The drug is slightly soluble at low pH and undergoes a substantial decrease in solubility when the solution pH increases above the pKa [5]. Despite a fairly long half-life, DLM requires twice daily dosing and must be taken with food to ensure adequate bioavailability [6].

A high percentage of marketed drugs and drug candidates have low aqueous solubility and consequently low bioavailability [7], [8]. As a result, there has been an increase in the number of amorphous solid dispersion formulations being used in commercial products [9]. However, drug crystallization is always a risk factor for these formulations. Whether or not crystallization occurs is a complex interplay of intrinsic drug properties that impact the crystallization tendency, formulation, processing, and storage conditions. Some drugs are simply inherently fast crystallizers, while other compounds are much easier to make and maintain in amorphous form.

Salt formation is a commonly employed strategy to alter the physicochemical properties of ionizable active pharmaceutical ingredients, including hygroscopicity, melting point and dissolution rate [8], [10], [11], [12]. Salt formation to produce crystalline salts is an extremely common approach to improve the developability of candidate drugs. The success of this strategy is highlighted by the observation that almost 50% of drugs marketed in 2007 were in the form of salts [11], [13]. Salt formation with acids that have a pKa at least 3 units lower than the base is considered important to achieve proton transfer and formation of a stable salt [14]. Widely used salts for weakly basic drugs include hydrochlorides, sulfonates and sulfates [11]. Between 2015 and 2019, there were five mesylate salts and eight tosylate salts in products approved by the United States Food and Drug Administration, suggesting that sulfonate salts are increasingly being utilized [15]. An important, but not very widely investigated observation, is that salt formation may alter the crystallization tendency of a drug [8], [16], [17]. Strong electrostatic interactions between the drug and counterions likely contribute to stabilizing the amorphous form of the drug via an enhanced glass transition temperature (*T_g_*) and reduced molecular mobility [17], [18], [19]. Furthermore, bulky counterions may provide steric hindrance to crystallization.

Combining salt formation with amorphous solid dispersions offers a potential strategy to inhibit the crystallization of rapidly crystallizing drugs, while maintaining the advantage of an amorphous formulation. Salt formation between basic drugs and acidic polymers has been reported in several studies [20], [21], [22], [23]. There are fewer studies where a drug salt is used in an amorphous solid dispersion formulation. Recently, Haser *et al*. [24] reported the *in situ* formation of a meloxicam-meglumine salt during hot melt extrusion. The same group has investigated salt formation between naproxen and meglumine, also during hot melt extrusion with polymers [25]. Given that salt formation can impact the crystallization tendency of a drug, ASDs containing the salt of a drug are of interest for compounds with a high propensity to crystallization.

**Fig. 1:**
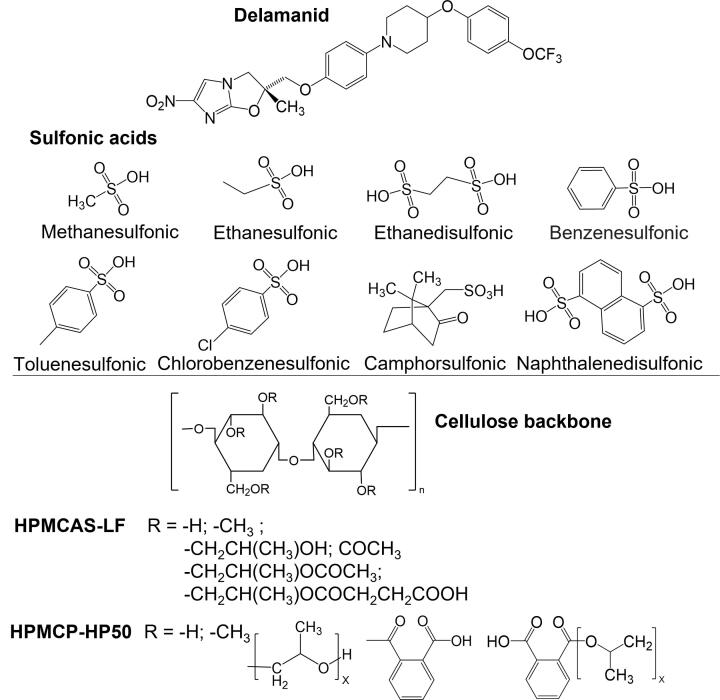
Chemical structures of DLM, sulfonic acids, HPMCAS-LF and HPMCP HP-50.

The goal of the current study was to improve amorphous stability and enhance the release of delamanid by developing amorphous solid dispersions containing a drug salt in combination with a polymer that is effective at inhibiting crystallization of the drug during preparation and dissolution. It was hypothesized that delamanid salts with sulfonic acids (Fig. 1) would be more resistant to solid-state crystallization than the corresponding amorphous free base due to their higher glass transition temperature. Moreover, by maintaining the amorphous nature of the drug salt in the ASDs, improved release performance would be achieved. Delamanid salts with the sulfonic acids shown in Fig. 1 were synthesized and characterized by evaluation of thermal transitions, X-ray powder diffraction patterns, hygroscopicity and chemical stability. Amorphous solid dispersions of delamanid free base and delamanid salts with either HPMCP or hydroxypropyl methylcellulose acetate succinate (HPMCAS) were prepared at different drug loadings (DLs) and subjected to similar characterization as for the neat amorphous salts. Release performance of the ASDs were evaluated using a two-stage dissolution test consisting of an initial low pH gastric stage followed by transfer to a higher pH medium corresponding to intestinal pH conditions.

## 2 Materials and methods

### 2.1 Materials

Delamanid (DLM) was obtained from Gojira Fine Chemicals, LLC (Bedford Heights, OH). Hydroxypropyl methylmellulose phthalate (HPMCP, HP-50 grade), hydroxypropyl methylcellulose acetate succinate (HPMCAS, LF grade) and hydroxypropyl methylcellulose (HPMC, substitution type 2910, grade 603) were procured from Shin-Etsu Chemical Co., Ltd. (Tokyo, Japan). Polyvinylpyrrolidone/vinyl acetate (PVPVA, Kollidon VA 64) was obtained from the BASF Corporation (FlorhamPark, NJ). 1,2-Ethanedisulfonic acid dihydrate (edisylate) and 4-chlorobenzenesulfonic acid (closylate) were purchased from Tokyo Chemical Industry Co. Ltd. (Tokyo, Japan). Methanesulfonic acid (mesylate), benzenesulfonic acid (besylate), and (1S)-(+)-10-camphorsulfonic acid (camsylate) were provided by Sigma-Aldrich (St. Louis, MO). Ethanesulfonic acid (esylate) and 1,5-naphthalenedisulfonic acid (napadisylate) were bought from Merck (Darmstadt, Germany).  P-toluenesulfonic acid (tosylate) was from Acros organic (Geel, Belgium). Cros-carmellose sodium and microcrystalline cellulose pH 101 (MCC) were sourced from FMC Biopolymer (Newark, DE). Magnesium stearate was supplied by Spectrum (New Brunswick, NJ). Eudragit L100 (Eud-L100) and silica, colloidal hydrated were from Evonik (Essen, Germany). Sodium starch glycolate was obtained from JRS Pharma (Rosemberg, Germany). Biorelevant simulated intestinal fluid powders (FaSSIF/FaSSGF and FaSSIF-V2) were purchased from Biorelevant (London, UK). Phosphate buffer solution (PBS) pH 6.5 (29 mM) and maleate buffer pH 6.5 (19 mM) were used to prepare FaSSIF V1 and FaSSIF V2 media, respectively. Dichloromethane (DCM), methanol (MeOH) and acetone were supplied by Fisher-Scientific (Pittsburg, PA).

### 2.2 Determination of drug solubility

The equilibrium solubility of crystalline DLM was determined by adding an excess amount of drug to the media of interest with stirring at 300 rpm at 37 °C for 48 h. The undissolved drug was removed by ultracentrifugation at 35,000 rpm (37 °C, 30 min) by an Optima L-100 XP ultracentrifuge (SW 41Ti rotor) (Beckman Coulter, Inc., Brea, CA). The supernatant was diluted in methanol to an appropriate concentration. The drug concentration was determined using an Agilent high performance liquid chromatography (HPLC) system (Agilent Technologies, Santa Clara, CA) with a C18 column (Zorbax Eclipse Plus, 4.6 × 250 mm, 5 μm, Agilent Technologies, Santa Clara, CA). The mobile phase comprised acetonitrile and water (75:25 by volume) at a flow rate of 1.5 mL/min and an injection volume of 20 µL. DLM was detected by UV absorbance at 320 nm.

The UV-extinction method [26] was used to determine the amorphous solubility of delamanid in fasted state simulated gastric fluid (FaSSGF), fasted state simulated intestinal fluid (FaSSIF) version 1 (V1) and version 2 (V2) (FaSSIF V1 and FaSSIF V2, respectively) and phosphate buffer solution (PBS) pH 6.5. The media pH ranged from 1.6 to 6.5 at 37 °C. Briefly, a stock solution of DLM in dimethyl sulfoxide (DMSO) was gradually added into the aqueous medium using a syringe pump (Harvard Apparatus, Holliston, MA) at a speed of 50–100 µL/min. The concentration of stock solution was adjusted to maintain the final organic solvent content below 1% (v/v). A 10 mm ultraviolet (UV) probe was used to monitor light scattering at non-absorbing wavelengths ranging from 400 to 450 nm by using a SI Photonics UV/vis spectrometer (Tucson, Arizona). The drug amorphous solubility was considered as the concentration where an abrupt increase in scattering was observed [27].

### 2.3 Nucleation induction time measurement

The nucleation induction time was determined to evaluate the impact of various polymers on the time until crystals could be detected. A SI Photonics UV/vis spectrometer (Tuscon, Arizona), coupled with a 10 mm probe was used for the measurements as described previously.[28] The increase in scattering resulting from the nucleation and growth of crystals was monitored at 30 s intervals by measuring the extinction at a non-absorbing wavelength (440 nm). The time until crystals were detected was evaluated at 37 °C at a concentration corresponding to the amorphous solubility of the drug in HCl solution, pH 1.6 or in PBS, pH 6.5. The ability to inhibit drug crystallization was determined with various polymers added to the PBS at a concentration of 300 μg/mL.

### 2.4 Salt preparation and characterization

The salt form of DLM was prepared using a Buchi Rotavapor-R (Newcastle, Delaware). DLM free base and sulfonic acid were added at drug-acid 1:1 molar ratio in a mixture of DCM and acetone (1:1 v/v) where alcoholic solvents were avoided to prevent the formation of alkyl esters during salt synthesis [29]. Solvents were removed by rotary evaporation at 40 °C. DLM salts were then kept under vacuum at room temperature overnight to remove residual solvents. DLM salts were characterized by ^1^H-nuclear magnetic resonance (NMR) spectroscopy to confirm salt formation. Their physical and chemical stability were monitored following storage at 40 °C/75% relative humidity (RH) by measuring several physicochemical properties, including crystallinity (by powder X-ray diffraction, PXRD), impurities (by ^19^F NMR spectroscopy), drug content (by HPLC as described above) and hygroscopicity (using dynamic vapor sorption analysis).

### 2.5 Dynamic light scattering

The size of DLM-rich droplets formed above the amorphous solubility was measured by dynamic light scattering (DLS) using a Nano-Zetasizer (Nano-ZS, Malvern Instruments, Westborough, MA). Nanodroplets were generated by adding a stock solution of delamanid in acetonitrile (10 mg/mL) into PBS pH 6.5 at 37 °C with stirring at 300 rpm. To evaluate the impact of polymers on nanodroplet stability, polymers were pre-dissolved in solution at a concentration of 300 μg/mL and drug was introduced at 50 μg/mL. Samples were monitored for up to 3 h after liquid–liquid phase separation was initiated. The impact of select polymers (HPMCAS and HPMCP) on nanodroplet formation was also evaluated as a function of drug concentration in the range of 2–100 μg/mL.

### 2.6 Preparation of DLM ASDs and DLM salt ASDs

ASDs of DLM free base and DLM salts were prepared using a Buchi Rotavapor-R (Buchi, Newcastle, DE). ASDs of DLM free base with HPMCAS and HPMCP were prepared by dissolving both components in DCM-MeOH (1:1 v/v), followed by evaporation. The drug-to-polymer ratio in the binary formulations is expressed as the mass ratio. DLM salt ASDs were prepared *in situ* by dissolving drug, polymer and acid in a mixture of DCM and acetone (1:1, v/v), avoiding MeOH to prevent the esterification reaction between sulfonic acids and alcohols [30], [31]. The drug-counterion was kept at a 1:1 molar ratio. After removing solvents using a rotary evaporator at 40 °C, ASD samples were kept under a vacuum at room temperature overnight, cryo-milled, and sieved to obtain the desired particle size fraction of 106–250 μm.

### 2.7 Powder X-ray diffraction (PXRD)

Crystallinity of ASDs and DLM salts was evaluated using a Rigaku Smartlab diffractometer (Rigaku Americas, The Woodlands, TX) equipped with a Cu–Kα radiation source and a D/tex ultradetector. Samples were added to glass sample holders, and powder patterns were recorded over the range of 4-40° 2θ at a scanning speed of 4° per min and a 0.02° step size with the voltage and current set to 40 kV and 44 mA, respectively.

### 2.8 Differential scanning calorimetry (DSC)

The glass transition temperatures (*T_g_*) of drug, polymers and ASD samples were determined using a TA Q2000 DSC equipped with an RCS90 refrigeration unit (TA Instruments, New Castle, DE). The temperature calibration was performed using indium and tin, while enthalpy calibration was conducted with indium. Samples (5–10 mg) were added to aluminum pans with a pinhole lid (Tzero pan, TA Instrument, DE). The sample was equilibrated at 0 °C and then heated from 0 to 210 °C at 10 °C/min and then cooled back down to 0 °C at 20 °C/min under a nitrogen flow of 50 mL/min. The heating and cooling cycle was repeated 3 times to remove residual solvent and thermal history, and the last cycle was used for analysis.

### 2.9 Infrared (IR) spectroscopy

The IR spectra of bulk polymers, drug and ASDs were collected in the attenuated total reflectance (ATR) mode using a Bruker Vertex 70 FTIR spectrometer (Billerica, MA) equipped with a Golden Gate ATR accessory (Specac, Orpington, Kent, UK). The spectrum of each sample was the average of triplicate runs of 32 scans and a resolution of 4 cm^−1^. The data were analyzed using OPUS software (version 7.2, Bruker, Billerica, MA).

### 2.10 Water sorption analysis

Water sorption profiles of DLM salts and ASDs were measured by using a SGA-100 symmetrical gravimetric analyzer (TA Instrument, New Castle, DE) at 37^°^ C. Samples (10–20 mg) were flushed with dry air for 3 h. After drying, samples were exposed to increasing relative humidity (RH) steps from 5 to 95% with a maximum step time of 180 min. Vapor sorption profiles are recorded as the equilibrium value (the weight change was below 0.001%/min over 5 min) at each RH.

### 2.11 ^1^H NMR and ^19^F NMR spectroscopy

Salt formation was confirmed by ^1^H nuclear magnetic resonance (NMR) spectroscopy and impurities in DLM salts following preparation and storage were detected by ^19^F NMR spectroscopy. Samples were dissolved in dimethyl sulfoxide-d6 (Cambridge Isotope Laboratories, Inc., Andover, MA) at a concentration of 20 mg/mL. All NMR spectra were acquired on a Bruker DRX 500 MHz spectrometer (Karlsruhe, Germany) equipped with a BBFO z-gradient probe operating at room temperature. For ^1^H NMR spectra, the spectral sweep width was 20 ppm, acquisition time was 1.6 sec and number of scans was 16. For ^19^F NMR spectroscopy, ^1^H was decoupled during acquisition, spectral sweep width was 50 ppm, acquisition time was 1.4 sec and the number of scans was 64.

### 2.12 Release testing

The ASDs were mixed with excipients and compressed to obtain tablets containing 5 mg DLM with respect to the free base. The tablet formulation is summarized in **Table S1**. All dissolution studies of DLM tablets were conducted in triplicate in single-stage or two-stage pH-shift condition using a Hanson Vision G2 Classic 6 dissolution system (Teledyne Hanson Research, Chatsworth, CA). For single-stage testing, the tablets containing the ASDs were added to 50 mL PBS, pH 6.5 and monitored for 1 h at 37° C, with 150 rpm of stirring. For pH-shift experiments, release in the acidic stage was conducted in 45 mL HCl solution pH 1.6 for 1 h, followed by addition of 5 mL concentrated PBS (pH 7.3) to adjust the pH of solution to pH 6.5 and drug release was monitored for an additional 30 min. An *in situ* Rainbow fiber optic ultraviolet spectrometer coupled with 10 mm fiber optic dip probes (Pion, Billerica, MA, USA) was used to monitor drug concentration over time. Second derivative analysis was applied to correct the spectral baseline and a calibration curve of area under curve (AUC) of the range 330–350 nm was used to calculate the drug concentration.

## 3 Results

### 3.1 Physicochemical properties of delamanid and delamanid ASDs

#### 3.1.1 Physicochemical properties and solubility of DLM free base

Physical properties of delamanid are summarized in Table 1. Delamanid is a weakly basic compound (pKa has been reported as 3.99 [32] or 4.3 [2]) and exhibits pH-dependent solubility (Table 1). Delamanid has crystalline and amorphous solubility values in FaSSGF of 17.6 ± 1.5 and 69.9 ± 2.6 µg/mL, respectively (Table 1). However, the drug is much less soluble at the higher pH value of the intestinal fluids. The crystalline and amorphous solubility values of DLM in PBS pH 6.5 were found to be 0.018 ± 0.003 and 0.76 ± 0.02 µg/mL, respectively. Higher solubility was noted in biorelevant media (FaSSIF V1 and V2), indicating that DLM undergoes solubilization in mixed bile salt/lecithin micelles.

**Table 1:** Physicochemical properties and solubility of delamanid free base.

**Parameter**	**Value**	
Log P	5.67 [33]; 6.14 [34]*	
pK_a_	3.99 [32]**; 4.3 [2]	
T_g_ (°C)	43.3 ± 0.4	
Melting temperature (°C)	196.1 ± 0.5	
Solubility *(µg/mL)*	*Crystalline*	*Amorphous*
FaSSGF	17.6 ± 1.5	69.9 ± 2.6
FaSSIF V1	0.52 ± 0.12	7.93 ± 0.11
FaSSIF V2	0.086 ± 0.009	3.11 ± 0.11
PBS pH 6.5	0.018 ± 0.003	0.76 ± 0.02
Induction time (min)		
FaSSGF (pH 1.6)	2.4 ± 0.5	
PBS (pH 6.5)	10.7 ± 1.2	

* predicted log P calculated by ALOGPS.

** predicted pKa.

Delamanid has T_g_ of 43.3 °C and shows a high tendency to crystallize in both the solid state and from aqueous solution. Amorphous delamanid could not be generated by cooling from the melt at a cooling rate as high as 50 °C/min (**Fig. S1**). Fast nucleation and crystal growth were also observed in both low and high pH environment with induction times (t_ind_) of 2.4 ± 0.5 and 10.7 ± 1.2 min, respectively, for a supersaturation corresponding to the amorphous solubility.

#### 3.1.2 Impact of polymers on induction time and nanodroplet size stability

**Fig. 2:**
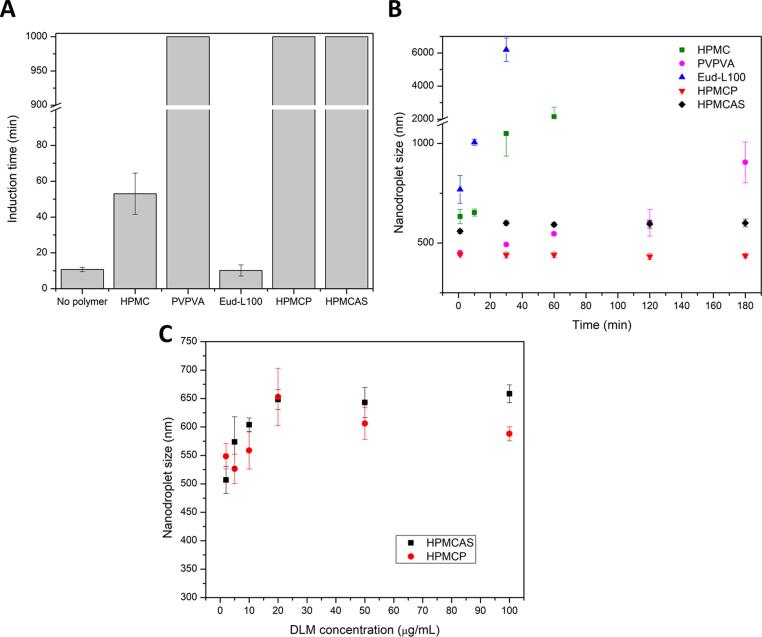
Impact of polymer on delamanid induction time and nanodroplet size stability: (A) Induction time in the presence of polymer at a concentration of 300 µg/mL. For PVPVA, HPMCP, and HPMCAS, no crystallization was observed for up to 1000 min; (B) Nanodroplet size as a function of time at an initial drug concentration of 50 µg/mL, and a polymer concentration of 300 µg/mL; (C) Droplet size as a function of drug concentration (polymer concentration of 1000 µg/mL, samples were measured 1 h after preparation).

Some polymers have been demonstrated as effective drug crystallization inhibitors in solution, with effectiveness attributed to the formation of intermolecular interactions with the drug [35], [36]. In this study, two neutral polymers (HPMC and PVPVA) and three weakly acidic polymers (Eud-L100, HPMCP and HPMCAS) were evaluated for their solution crystallization inhibition effectiveness at a polymer concentration of 300 µg/mL (Fig. 2**A**). Induction times were measured in PBS pH 6.5 where both neutral and enteric polymers are soluble. At a supersaturation corresponding to the amorphous solubility of delamanid (0.76 ± 0.02 µg/mL), the drug showed rapid nucleation and crystal growth (induction time of 10.7 ± 1.2 min). For the neutral polymers, PVPVA showed good inhibition of drug crystallization where the supersaturation was maintained for more than 1000 min, whereas in the presence of HPMC, crystallization commenced in about 50 min. For the weakly acidic polymers, Eud-L100 was ineffective as the induction time was similar to that observed in the absence of the polymer (10.2 ± 3.1 min). In contrast, HPMCP and HPMCAS were highly effective at inhibiting crystallization of delamanid with no crystallization observed within 1000 min.

Besides inhibiting crystallization, polymers can also stabilize the drug-rich droplets formed when the amorphous solubility is exceeded [37]. At a concentration of 50 µg/mL (>50 times higher than delamanid amorphous solubility), drug-rich nanodroplets were formed but crystallized immediately in the absence of polymer. The polymers delayed crystallization, allowing nanodroplet size to be probed. The initial size of the nanodroplets in the presence of different polymers followed the trend: HPMCP ∼ PVPVA < HPMCAS < HPMC < Eud-L100 (Fig. 2**B**). However, nanodroplets were short-lived in Eud-L100 and HPMC solutions, where crystallization occurred at short time frames, with rapid increase in the size of the scattering species. PVPVA inhibited crystallization, but nanodroplets gradually ripened, increasing in size with time. On the other hand, there was no notable change in the size of nanodroplets in HPMCP and HPMCAS solutions over the 3-h period monitored. Furthermore, these polymers were able to stabilize delamanid nanodroplets even when the drug concentration was increased to 100 µg/mL (Fig. 2**C**). Based on these observations, HPMCP and HPMCAS were selected to prepare DLM ASDs.

#### 3.1.3 Characterization of DLM free base ASDs

**Fig. 3:**
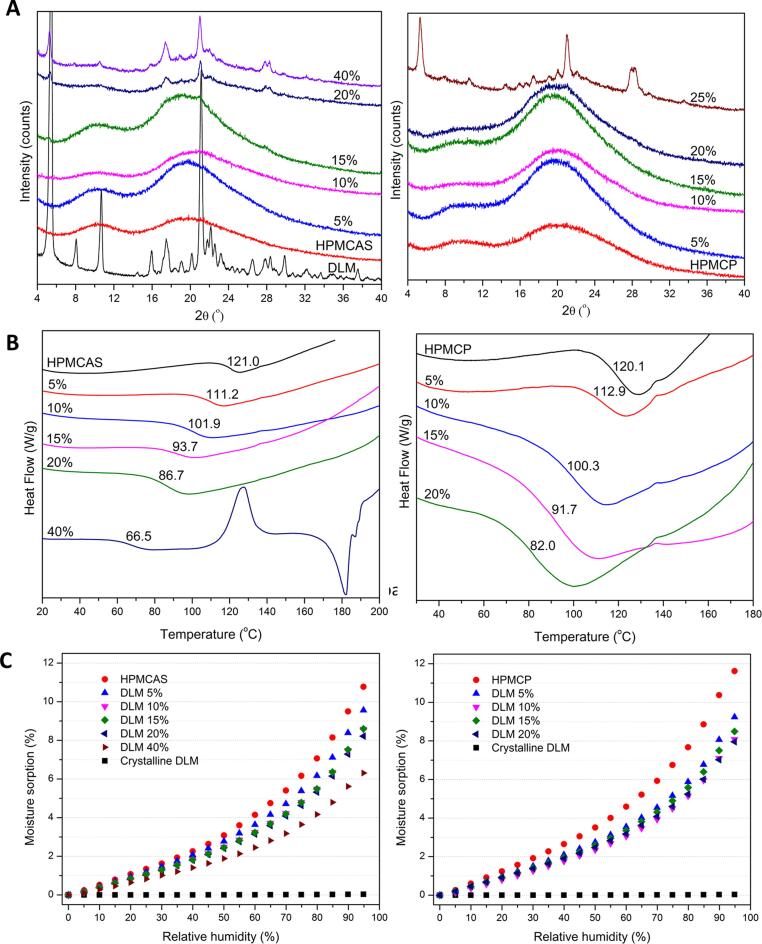
Physical properties of DLM ASDs with HPMCAS and HPMCP as a function of drug loading: (A) PXRD patterns; (B) DSC thermograms showing T_g_ and (C) water sorption profiles*.*

ASDs of DLM free base prepared with either HPMCAS or HPMCP by rotary evaporation were found to contain residual crystallinity immediately following preparation, for DLs > 10–15%. PXRD data in Fig. 3**A** shows evidence of drug crystallinity at 15% drug loading for HPMCAS and 20% drug loading for HPMCP. Delamanid has a much lower T_g_ (43.3 °C) than the polymers (121.0 °C and 120.1 °C for HPMCAS and HPMCP, respectively) and DLM was an effective plasticizer; the T_g_ values of the ASDs were lowered with increasing amounts of drug (Fig. 3**B**). Crystalline delamanid free base was non-hygroscopic with very low water sorption (Fig. 3**C**). In contrast, the neat polymers showed higher water sorption with about 11–12% water being absorbed at 95% RH. The hygroscopicity was reduced with increasing drug loading in the ASDs. No specific intermolecular interactions between drug and polymers were detected by infrared spectroscopy (**Fig. S2**).

#### 3.1.4 Drug release from DLM free base ASDs

Initial release studies with ASD powders suggested wetting issues with powder floating on the top of the dissolution vessel; therefore, release studies were performed with tablets. No wetting issues were observed with the tablets which showed rapid disintegration and dispersion of the resultant particles. Due to the fact that similar release profiles were observed for biorelevant media versus buffer (**Fig. S3**), all release tests were performed in buffer.

**Fig. 4:**
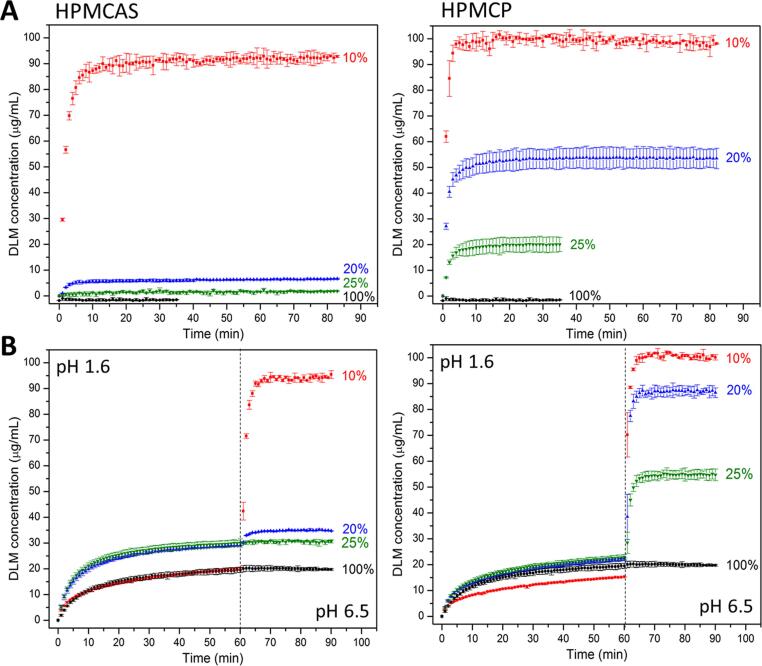
Drug release profiles of DLM ASDs as a function of drug loading in (A) PBS pH 6.5 and (B) pH-shift experiment. Dashed line indicates the shifting of pH from 1.6 to 6.5.

Drug release profiles for DLM free base ASD tablets are summarized in Fig. 4. The maximum theoretical drug concentration for 100% release is 100 µg/mL, approximately 100 times the amorphous solubility and the dissolution conditions are thus highly non-sink with respect to both the crystalline and amorphous solubilities. For crystalline drug, no release was detected in PBS pH 6.5, while a concentration of around 20 µg/mL was observed in the pH shift experiment, due to the initially higher solubility in a low pH environment.

The ASD formulation with the enteric polymers considerably improved the drug release, especially at low drug loadings, where the drug was maintained in the amorphous state in the ASD matrix following preparation. Complete release was observed in both the single-stage test and the pH-shift experiment when the drug loading was only 10%, for ASDs with both polymers. Importantly, both enteric polymers were able to prevent crystallization from the supersaturated solution formed following drug release.

However, the higher drug loading ASDs, which contained residual crystallinity, based on the PXRD analysis (Fig. 3**A**), performed poorly. Presumably, the DLM crystals present in the ASD were able to grow either in the hydrated matrix, or after release into solution, reducing the achievable supersaturation and leading to poor release, as observed in other systems [38], [39]. For HPMCP, the drug release dropped to 50% for the 20% DL ASD, which further decreased to 20% for the 25% DL. The decrease in release on increasing the DL from 20 to 25% DL was consistent with the greater extent of crystallinity in the latter sample (Fig. 3**A**). Similarly, ASDs with HPMCAS also showed a notable decrease of drug release when the drug loading increased from 10% to 20%, corresponding the greater tendency of these ASDs to crystallize during preparation. Interestingly, the drug release rate was rapid for all systems and reached a plateau level within 5–10 min. In general, comparing between the two polymers, HPMCP ASDs exhibited better release than HPMCAS ASDs at comparable DLs.

#### 3.1.5 Dissolution performance of Deltyba^TM^ tablet

**Fig. 5:**
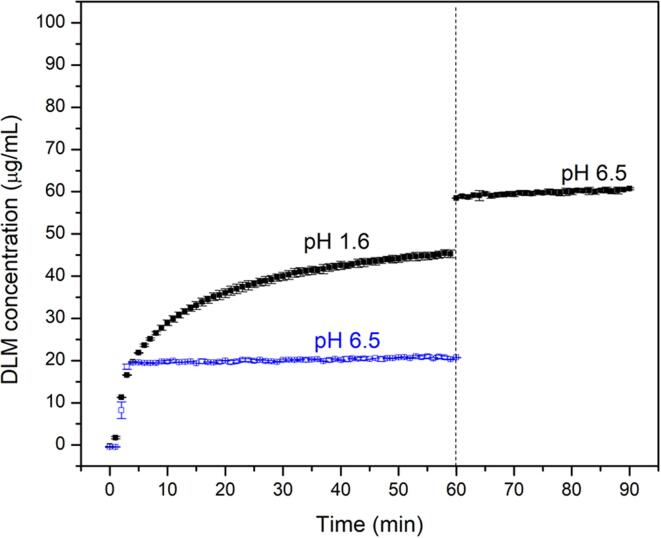
Dissolution profile of Deltyba^TM^ tablet in PBS pH 6.5 (blue) or pH-shift experiment (black). Dash line indicates pH shift from acidic into intestinal environment. (For interpretation of the references to color in this figure legend, the reader is referred to the web version of this article.)

The marketed product (Deltyba^TM^) contains an ASD of delamanid with HPMCP, prepared by spray drying. The estimated drug loading relative to polymer is ∼20–25%, based on the results of reverse engineering experiments (as described in the Appendix A). Fig. 5 shows that there is incomplete release from the reference product in both single stage and pH-shift experiments. A maximum drug concentration of about 20 µg/mL was obtained in PBS pH 6.5, which was similar to the DLM ASD with HPMCP at a 25% DL (Fig. 4**A**). In the pH-shift dissolution measurement, higher drug release was noted in the acid stage (about 45 µg/mL) followed by a small extent of additional release upon moving to higher pH environment, although release was incomplete.

### 3.2 Salt formation and characterization of DLM salts

Salts of DLM with a variety of sulfonic acids (molecular properties of counterions are summarized in **Table S2**) were evaluated to identify the best candidates for incorporation into ASDs.

The solution-state ^1^H NMR spectral comparison of DLM free base and DLM edisylate reveals that salt formation has a substantial impact on the ^1^H spectra of DLM. The peaks assigned to protons of the piperidine ring of the free base significantly shifted downfield in the salt due to the protonation of the tertiary amine group following interaction with the sulfonic acid that changed the electron density of the adjacent atoms (**Fig. S4**). In contrast, almost no change was observed for protons near the imidazole ring (**Fig. S4**). This suggested that the piperidine ring is the site of ionization for salt formation. Further evidence of salt formation, specifically protonation of a nitrogen group, is provided in **Fig. S5,** with the appearance of a very broad and intense infrared peak in the range from 2770 to 2380 cm^−1^ (maximum peak at 2519 cm^−1^) in sulfonate salts, which was assigned to the characteristic tertiary N^+^-H stretching vibration [40]. Methylene groups next to the nitrogen atom in the tertiary amine group of the free base resulted in bands at 2816 cm^−1^ which were absent from the salt spectra. The protonation of the tertiary amine group increased the C-H binding constant, displacing these bands to a higher frequency region, causing them to overlap with other bands in this spectral range (such as C-H stretch aromatic and aliphatic) [41], [42], [43], [44].

**Fig. 6:**
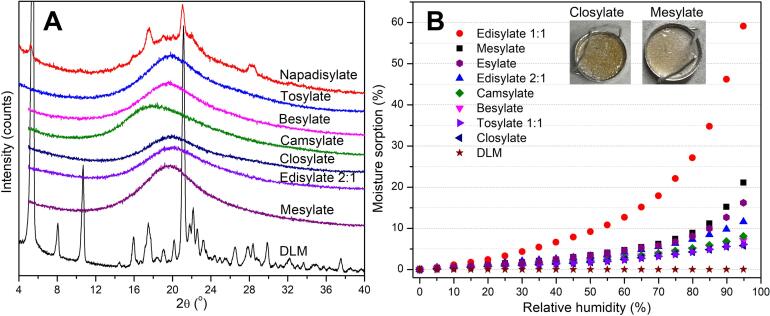
Physical properties of DLM sulfonate salts (A) XRPD diffractograms and (B) Water sorption profiles.

Fig. 6A shows partial crystallization for the DLM-napadisylate salt while all other DLM sulfonate salts could be readily produced in amorphous form. Salt formation resulted in an increased hygroscopicity relative to DLM free base (Fig. 6**B**). The highest moisture sorption was observed for the mesylate salt with approximately 21% water uptake at 95% RH. In addition, several salts appeared to undergo liquefaction (presumably due to a high extent of plasticization by water) following storage at 40 °C/75% RH including mesylate, esylate, tosylate, besylate and closylate salts (Fig. 6**B** and Table 2). Less hygroscopicity was noted for aromatic sulfonic salts, which can be attributed to the more lipophilic properties of these counterions.[45] Furthermore, excess counterion in the DLM-edisylate (1:1) salt led to increased water sorption (59% at 95% RH). The induction times (t_ind_) at the amorphous solubility for the DLM salts and free base were similar as expected; around 2 min in FaSSGF and less than 20 min in PBS pH 6.5 (Table 2).

**Table 2:** Physicochemical properties of DLM sulfonate salts.

**Property**	**Besylate**	**Camsylate**	**Closylate**	**Edisylate (2:1)**	**Esylate**	**Mesylate**	**Tosylate**	**Napadisylate (2:1)**
%DLM in the salt	77	70	74	85	83	85	76	79
Amorphous	Y	Y	Y	Y	Y	Y	Y	N
T_g_ (^o^C)	85.1	97.9	90.1	117.8	83.7	86.2	90.2	N/A
t*_i_*_nd_ in FaSSGF (min)	1.1	1.3	1.1	0.6	1.0	1.1	1.0	N/A
t_ind_ in PBS pH 6.5 (min)	16.6	14.7	14.1	16.0	19.7	15.7	18.5	N/A
Water sorption (%) at 95 % RH	7.1	8.1	5.8	11.6	16.2	21.1	6.1	N/A
**Stability *(2 weeks, 40 °C/RH 75%)***								
Liquefaction	+	–	–	–	+	+	+	N/A
Amorphous	Y	Y	Y	Y	Y	Y	Y	N/A
Impurity (^19^F NMR) (%)	0.5	2.0	2.3	1.0	0.7	0.3	1.0	N/A
Drug content reduction (%)	0.5	6.1	3.1	0.8	N/A	2.8	2.1	N/A

t*_ind_* induction time; *N/A not applicable*.

Salt formation with sulfonic acids at a drug-counterion of 1:1 molar ratio led to an increase in *T_g_* of DLM salts as compared with DLM free base (Table 2). The highest *T_g_* compound was the DLM-edisylate (2:1) salt (117.8 °C). However, the presence of excess counterion resulted in a notable decrease in *T_g_* (53.9 °C for DLM-edisylate (1:1) salt).

The stability of DLM sulfonate salts was monitored for 2 weeks under open dish accelerated storage conditions of 40 °C/75% RH. All initially amorphous DLM salts were physically stable over this time period, and no peaks were detected from the PXRD patterns (**Fig. S6**). Impurities and drug content were evaluated by ^19^F NMR spectroscopy and HPLC analysis. Impurity peaks were observed in the ^19^F NMR spectra of DLM-closylate, DLM-camsylate, DLM-edisylate (2:1) and DLM-tosylate salts, with DLM-closylate and DLM-camsylate salts showing the largest relative peak areas (2.3% and 2.0%, respectively) (Table 2 and **Fig. S7A**). Similarly, drug content was found to decrease in these four salts, in particular DLM-closylate and DLM-camsylate (**Fig. S7B**). Based on the physicochemical properties and stability of the various salts, four salts were selected for ASD formation, namely the besylate, edisylate (2:1), mesylate and tosylate salts.

### 3.3 Characterization of DLM salt ASDs

#### 3.3.1 Moisture sorption and T_g_ of DLM salt ASDs

**Fig. 7:**
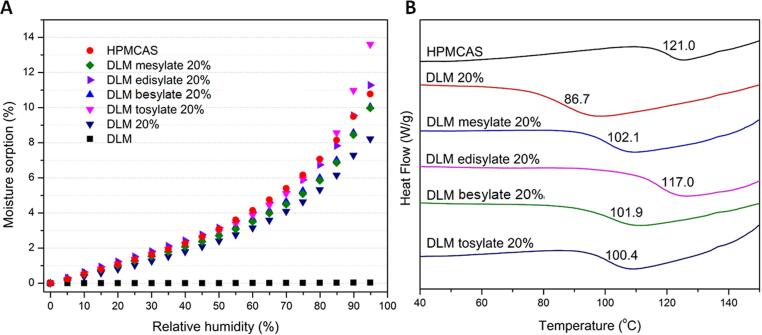
(A) Water sorption profiles and (B) glass transition temperature of DLM salt ASDs with HPMCAS at 20% drug loading.

DLM salt ASDs were prepared *in situ* with HPMCAS-LF and HPMCP HP-50 by rotary evaporation. In general, DLM salt ASDs showed higher water sorption than ASDs containing DLM free base. For example, for a 20% DL HPMCAS ASD, the water content for the free base was about 8% at 95% RH (Fig. 3C), while DLM salt ASDs had water contents in the range of 10–14% (Fig. 7**A**). ASDs containing salts exhibited higher T_g_s than ASDs with the corresponding free base. The T_g_ of the 20% DL free base ASD with HPMCAS was ∼87 °C (Fig. 3**B**) while the T_g_s of DLM salt ASDs were above 100 °C (Fig. 7**B**). The DLM-edisylate ASD yielded the highest T_g_ of 117.0 °C, which is consistent with the high T_g_ of the neat salt (Table 2**)**.

#### 3.3.2 Release profiles of DLM salt ASDs

**Fig. 8:**
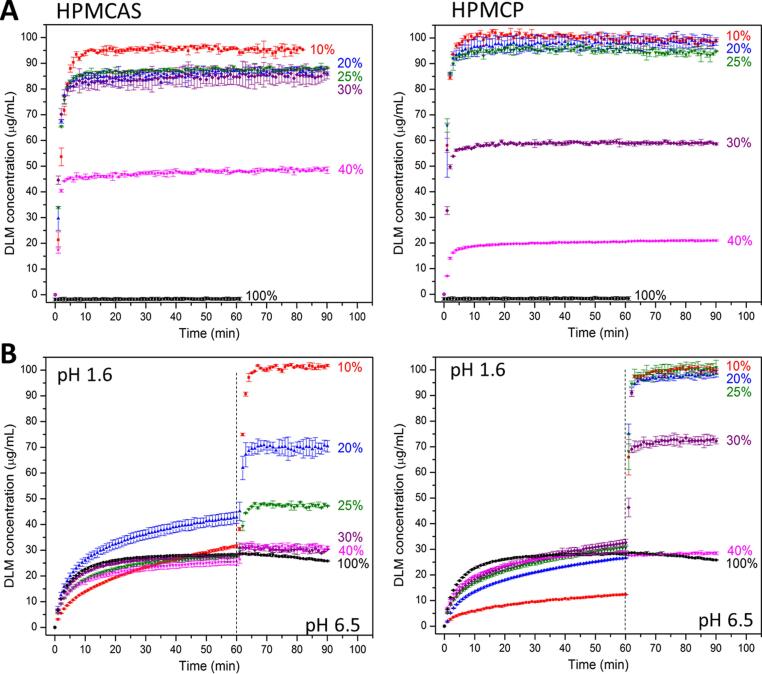
Drug release from DLM-edisylate ASDs as a function of drug loading in (A) PBS pH 6.5 and (B) pH-shift experiments. Dashed lines indicate the pH change from pH 1.6 to pH 6.5.

Drug release profiles from ASDs as a function of drug loading were evaluated with the DLM-edisylate salt. The neat DLM-edisylate showed no release in PBS pH 6.5 (Fig. 8**A**). In an acidic medium, the release of neat DLM-edisylate (Fig. 8**B**) was slightly higher than that observed for DLM free base (Fig. 4**B**) but was lower than the drug amorphous solubility.

DLM-edisylate ASDs with 10–40% DL showed enhanced release relative to DLM free base ASDs (Fig. 4). In PBS pH 6.5, ASDs with HPMCAS exhibited nearly complete release for DLs from 10 to 30%, while the DL boundary of good release for ASDs of HPMCP was 25% (Fig. 8**A**). For pH-shift experiments, HPMCAS ASDs were found to release drug to a greater extent in the acidic media relative to the corresponding HPMCP ASDs (Fig. 8**B**). However, under fasted-state intestinal pH conditions, drug release was better from HPMCP ASDs. For HPMCAS ASDs, only the 10% DL system showed almost complete release in the two-stage dissolution test, while for HPMCP ASDs with DLM-edisylate, good release was observed for DLs as high as 25%. In addition, stable drug-rich nanodroplets were generated in the dissolution medium at various DLs (**Fig. S8**). In general, ASDs of HPMCP yielded smaller drug-rich droplets than those with HPMCAS, similar to findings from the solvent-shift experiments (Fig. 2**)**.

**Fig. 9:**
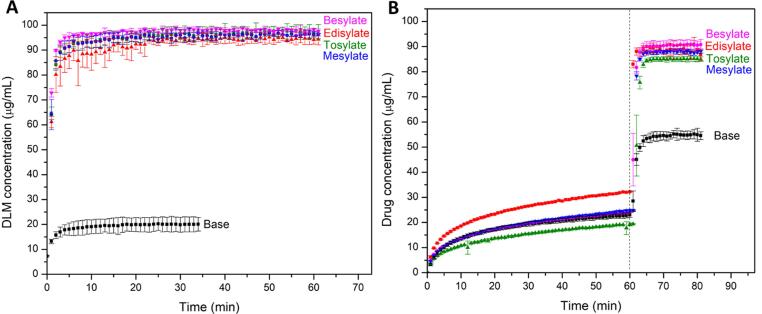
Release profiles of DLM sulfonate salt ASDs with HPMCP at a 25% drug loading in (A) PBS pH 6.5 and (B) pH-shift experiment. Dashed lines indicate the pH change from pH 1.6 to pH 6.5.

Fig. 9 provides a summary of the counterion impact on drug release, at a 25% DL with HPMCP. All four sulfonate salts showed a similar pattern with near-complete release in the single-stage test in PBS pH 6.5 medium (Fig. 9**A**). In acidic medium, the concentration released ranged from 20 to 35 µg/mL, with the highest amount observed for the DLM-edisylate ASD. Despite differences in the extent of drug release in the gastric stage, all formulations exhibited rapid and essentially complete release when the pH was increased from 1.6 to 6.5 (Fig. 9**B**).

## 4 Discussion

### 4.1 Salt formation and crystallization tendency

Delamanid is a poorly water-soluble compound with a high tendency to crystallize. Several factors might impact glass forming ability and crystallization tendency, including the free energy difference between the crystalline and amorphous states, molecular mobility of the amorphous material, molecular weight and complexity, and differences in intermolecular interaction patterns in the crystal versus amorphous materials [46]. It has been proposed that the solubility difference between the amorphous (*C_a_*) and crystalline (*C*_s_) forms of a drug compound at a given temperature (*T*) is directly related to the free energy difference (Δ*G*) between the two forms: Δ*G* = -*RT* ln(*C_a_/C_s_*) (1), where *R* is the gas constant.[47].

Δ*G* can be estimated from the Hoffman equation as following [48]:

(2) $$\Delta G=-\frac{\Delta H_{f}\left(T_{m}-T\right)T}{T_{m}^{2}}$$

in which *ΔH_f_* is the enthalpy of fusion measured at the melting point (*T_m_*).

By using the *ΔH_f_* and *T_m_* values obtained from the DSC thermogram (**Fig. S1**), the solubility ratio of amorphous DLM relative to its crystalline counterpart at 37 °C was calculated to be 31.8, which is in reasonable agreement with the experimental ratio of 42.3 (Table 2**)**.

Delamanid crystallizes during cooling from the melt at 50 °C/min and hence is a poor glass former (Class I compound).[46] Glassy delamanid can be obtained by quenching the melt in liquid N_2_, yet devitrifies upon heating to just above the *T_g_* (**Fig. S9**). The poor glass stability of delamanid makes it challenging to prepare crystal-free ASDs via rotary evaporation, for drug loadings > 10%. The solvent evaporation rate plays an important role in determining if material can be trapped in a metastable state before nucleation and crystal growth can occur [46], [49]. Clearly, the solvent evaporation rate using rotary evaporation was not fast enough to prevent crystal nucleation and growth, even in the presence of a polymer, for delamanid DLs of interest, leading to ASDs with residual crystallinity (Fig. 3**A**). It is likely that ASD formation methods that achieve faster evaporation rates, e.g. spray drying, could lead to crystal-free ASDs at higher drug loadings.

Salt formation led to increased glass transition temperatures and enhanced the stability of amorphous salts against crystallization. Neat amorphous DLM salts could be readily prepared by solvent evaporation and showed remarkable stability to crystallization when exposed to harsh storage conditions, with the exception of the napadisylate salt. This strategy was thus highly successful in enabling higher DL ASDs to be prepared with no detectable residual crystallinity, demonstrating that the low crystallization tendency of the salts translated to the ASD during preparation and storage. Historically, salt formation has been used to improve the crystallinity of a given drug. There are relatively few studies on improving glass forming ability through salt formation [10], [19], [50], although just as counterions can promote formation of an ordered crystal lattice, it can be anticipated that they can equally effectively disrupt the lattice, depending on the nature of the counterion, and the site of ionization. For delamanid, the site of ionization is the piperidine ring. We can speculate that salt formation disrupts the ability the long, thin delamanid molecules to line up to form an ordered crystal structure, although unfortunately, the single crystal structure of this molecule has not been obtained to date.

### 4.2 Factors impacting delamanid release from ASDs

Amorphous solid dispersions are an effective strategy to generate a supersaturated solution of the drug [51], [52]. The polymers used to form the ASD matrix play a critical role in kinetically stabilizing the supersaturated solution generated upon drug release and are thought to inhibit drug crystallization via the formation of non-specific interactions between the drug and polymers [35], [36]. However, not all polymers are equally effective at maintaining supersaturation. Herein, PVPVA, HPMCAS and HPMCP were found to be effective solution crystallization inhibitors, extending the induction time from a few minutes to up to >12 h (Fig. 2**A**). However, size enlargement of DLM-rich droplets occurred in presence of PVPVA, as observed for other systems [37]. Given that drug-rich nanodroplets are thought to be important in improving oral bioavailability for some drugs [53], [54], where nanodroplet size may play a role [55], PVPVA shows disadvantages for ASD formulations relative to HPMCAS and HPMCP. Indeed, the high sensitivity of delamanid to the polymer used to form that ASD is highlighted in **Fig. S10**, where the enteric polymers facilitate drug release to a much greater extent than the two neutral polymers evaluated [56].

Although HPMCAS and HPMCP are very good at inhibiting crystallization from highly supersaturated delamanid solutions, these polymers are less effective at preventing crystallization of DLM free base during ASD fabrication via solvent evaporation, leading to residual crystallinity in ASDs above a threshold drug loading. Residual crystals directly result in lost solubility advantage and, for some drugs, can act as seeds for additional crystal growth during matrix hydration and drug release [38], [39]. For delamanid, once any level of residual crystallinity could be detected in the ASD, release was incomplete during dissolution testing (Fig. 3**A**). This can be explained by the growth of crystals formed during the manufacturing process which competes with the dissolution of the surrounding amorphous material [39]. Further, the low release indicates that the polymers are unable to substantially delay crystal growth in hydrated conditions, making residual crystallinity extremely detrimental to ASD performance. This is different from the case of indomethacin, where residual crystals were found to be effectively poisoned by the ASD polymer [39], but similar to tacrolimus [38], [57], and bicalutamide [58] dispersions with residual crystallinity, where trace crystallinity substantially impacted release extent. Interestingly, HPMCAS ASDs appeared to contain a higher extent of residual crystallinity than the corresponding HPMCP ASDs at a comparable drug loading (20% DL, Fig. 4), which in turn translated to a lower extent of release for the former systems. Notably, the marketed product (Deltyba^TM^) contains a spray dried ASD with HPMCP, with an estimated drug loading relative to polymer of 20–25%. Higher drug release is observed in acidic medium, relative to our formulation. However, following transfer to pH 6.5 media, the maximum extent of release is approximately 60% from both Deltyba^TM^ tablets (Fig. 5**)** and our 25% DL free base HPMCP ASD formulation (Fig. 4**)**. This may indicate a similar failure mechanism for Deltyba^TM^ ASDs as for the 25% DL ASD but would require further investigation.

Preparation of amorphous solid dispersion of salts has been suggested as a promising strategy to combine the benefits of amorphization and salt formation for improved dissolution and stability against crystallization during storage [10]. For delamanid, using certain sulfonic acid salts in the fabrication of ASDs led to a clear improvement in the attainable drug loading where crystal-free systems could be obtained immediately following preparation. The formation of completely amorphous ASDs with the salts and HPMCP then translates into improved release for both single-stage and two-stage dissolution (25% DL, Fig. 8). As for the ASDs of free base, HPMCP solid dispersions with the salts generally yield improved release profiles relative to ASDs with HPMCAS.

Interestingly, different patterns of release are observed for single-stage and two-stage dissolution. Several salt ASDs show diminished release performance when incubated in a low pH environment prior to shifting to higher pH media. Due to the fact that the polymers are insoluble at low pH, this observation may suggest physical instability of the drug remaining in the ASD matrix during the acid immersion stage. Given the expected variation in gastric pH within patients as a function of fed and fasted state, and inter-patient pH variations arising from factors such as age or concomitant mediations (e.g. proton pump inhibitors), as well as general variability, further studies of pH effects should be carried out. Importantly, even higher drug solubility in biorelevant media was noted, although there was no difference in release profiles in buffer versus in FaSSIF (**Fig. S3**). The results presented herein also suggest that ASD formulations should be subjected to biorelevant dissolution testing conditions, at least in terms of pH changes, especially for systems employing enteric polymers and ionizable drugs.

## 5 Conclusions

Crystallization is a major failure mechanism for ASDs. Herein, we have demonstrated that drug crystallization tendency can be manipulated via salt formation. Amorphous delamanid was difficult to prepare and underwent rapid recrystallization. In contrast, several sulfonate salts remained amorphous for extended periods of time when exposed to stress storage conditions. By *in situ* salt formation during ASD manufacture, drug crystallization was prevented at higher drug loadings and release profiles were improved. In general, delamanid salt ASDs with HPMCP outperformed those fabricated with HPMCAS during release testing. Salt formation, combined with ASD formation, thus provides a dual strategy to address dissolution and solubility challenges with poorly soluble compounds that are difficult to formulate using a single enabling strategy.

## Declaration of Competing Interest

The authors declare that they have no known competing financial interests or personal relationships that could have appeared to influence the work reported in this paper.
