# Supplementary material for: Combining enabling formulation strategies to generate supersaturated solutions of delamanid: In situ salt formation during amorphous solid dispersion fabrication for more robust release profiles
Source: Eur J Pharm Biopharm. 2022 May;174:131–43. doi: 10.1016/j.ejpb.2022.04.002 (PMC9084191; doi:10.1016/j.ejpb.2022.04.002)
Supplement: Supplementary data 1 [file mmc1.docx]

**Supplementary material**

**Combining enabling formulation strategies to generate supersaturated solutions of delamanid: *in situ* salt formation during amorphous solid dispersion fabrication for more robust release profiles**

Tu Van Duong^1, §^, Hanh Thuy Nguyen^1, §^, and Lynne S. Taylor^1, *^

^1^ Department of Industrial and Physical Pharmacy, College of Pharmacy, Purdue University, West Lafayette, Indiana 47907, United States

^§^The authors contribute equally.

^*^ Corresponding author. E-mail: lstaylor@purdue.edu. Tel: +1 (765) 496-6614. Fax: +1 (765) 494-6545.

**Table S1. Formulation of DLM tablet.**

| **Components** | **Amount (mg)** |
| --- | --- |
| DLM free base or salt or ASDs of DLM | Equiv. 5 mg DLM |
| Sodium starch glycolate | 4 |
| Carmellose calcium | 4 |
| Silica, colloidal hydrated | 0.6 |
| Magnesium stearate | 0.6 |
| MCC PH 101 | q.s. 75 |

*
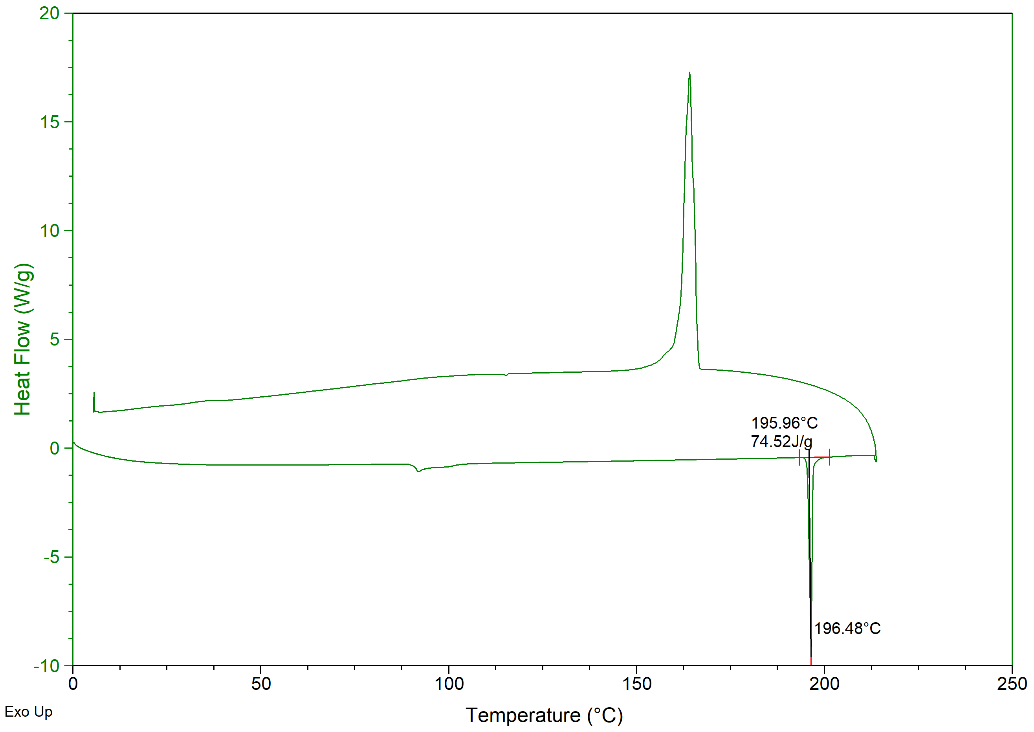
*

**Fig. S1. Differential scanning calorimetry thermograms showing rapid crystallization of amorphous delamanid at a cooling rate of 50^o^C/min from the melt.**

**
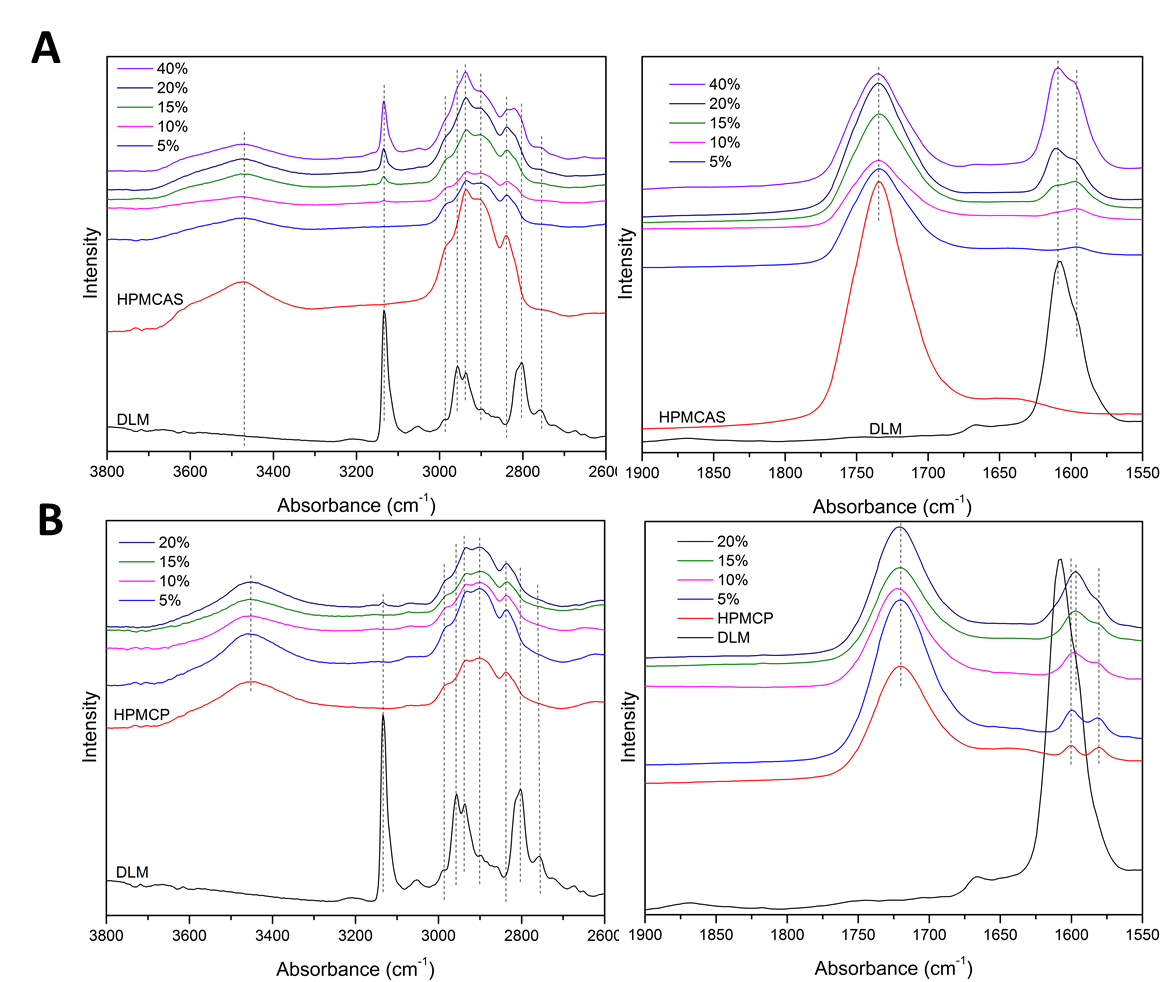
**

**Fig. S2. Infrared spectra of DLM ASDs with (A) HPMCAS and (B) HPMCP. The percentage indicates the drug loading in wt. %.**

*
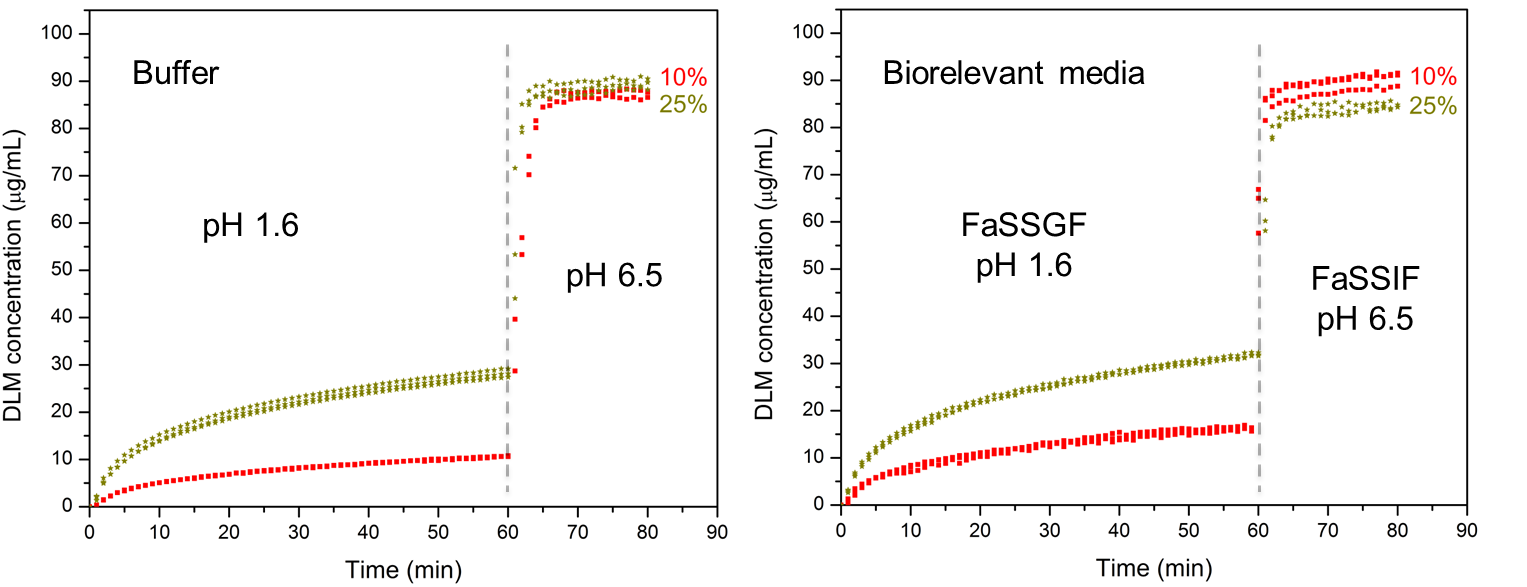
*

**Fig. S3. Dissolution profile of DLM-edisylate ASDs with HPMCP in buffer and biorelevant media.**

**Appendix A: Reverse engineering**

Deltyba^TM^ tablet (average weight of 530 mg) contains 50 mg delamanid and various excipients, including hypromellose phthalate (HPMCP), povidone, tocopherol, lactose monohydrate, MCC, sodium starch glycolate, carmellose calcium, magnesium stearate, silica, hypromellose, macrogol 8000, titanium dioxide, talc and iron oxide yellow. One Deltyba^TM^ tablet was crushed and dissolved in 10 mL of DCM:MeOH (1:1) mixture to dissolve the HPMCP. This was then filtered using a 0.45 µm PTFE filter. 50 µL of this solution was then added to 10 mL of 50 mM pH 6.8 buffer and stirred to evaporate the DCM. The solution became turbid most likely due to the fact that the organic solvent also dissolved delamanid (this could contribute slightly to the color obtained since delamanid changes color in acidic media). The solution was filtered through a 0.45 µm PTFE syringe filter and 2 mL of this filtrate added to a different vial. To this was added 50 µL of phenol and 5 mL of concentrated sulfuric acid. The solution was kept at room temperature for 10 minutes to allow the color to develop, and the absorbance was determined at 490 nm. Standard solutions of HPMCP HP-55 were also prepared, and a standard curve was made using the same protocol.

Alternatively, one Deltyba^TM^ tablet was dissolved in 20 mL of 0.1 N HCl for 2 h and then filtered. The residue was then dissolved in 200 mL of 50 mM pH 6.8 buffer. This solution was then filtered through a 1 µm nylon filter and 1 mL was diluted to 10 mL. To 2 mL of this solution, 50 µL of phenol as added and then 5 mL of concentrated sulfuric acid was added to give a yellow color. The solution was left at room temperature for 10 min and then evaluated using a Cary UV spectrometer at 490 nm.

**Table S2. Chemical properties of sulfonic acids used to prepare DLM sulfonate salts.**

| **Sulfonic acid** | **MW (g/mol)** | **Chemical structure** | **pK_a_^α^** | **Frequency of use in commercial products (%) [1]** |
| --- | --- | --- | --- | --- |
| Methanesulfonic | 96.11 | 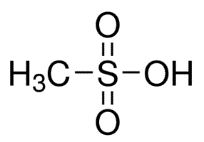 | -1.92 [2]  or -1.20 [3] | 3.2 |
| Ethanesulfonic | 109.13 | 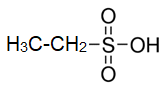 | -1.68 [2]  or -2.05 [3] | 0.13 |
| Toluenesulfonic | 172.20 | 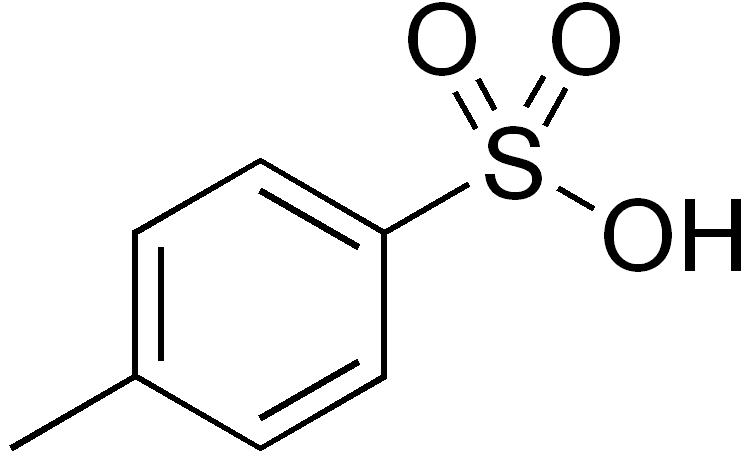 | -1.34 [3] | 0.39 |
| Benzenesulfonic | 158.18 | 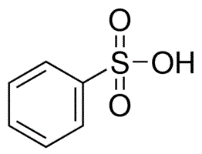 | -2.8 [2]  or 0.70 [3] | 0.26 |
| Ethanedisulfonic | 190.18 | 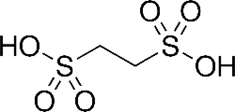 | -2.1 [3, 4] | 0.20 |
| Naphthalenedisulfonic | 288.30 | 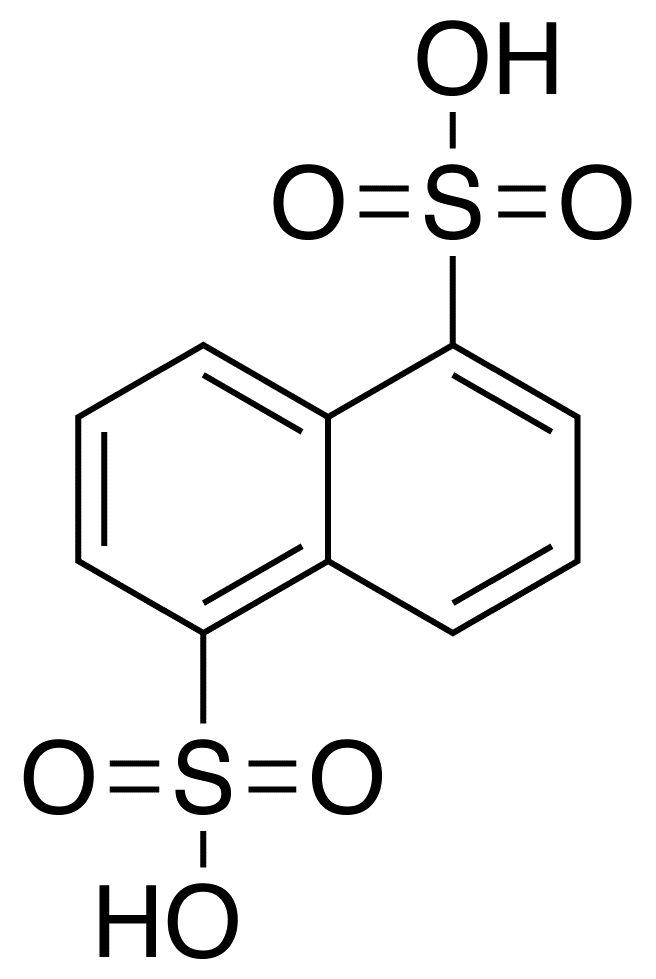 | 0.17 [3, 4] | 0.20 |
| Chlorobenzenesulfonic | 192.62 | 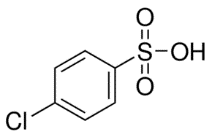 | -0.61 [5] | 0.07 |
| Camphorsulfonic | 232.29 | 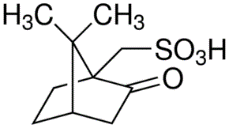 | 1.2 [6] | 0.59 |

*^α^ Value of the strongest acidity*


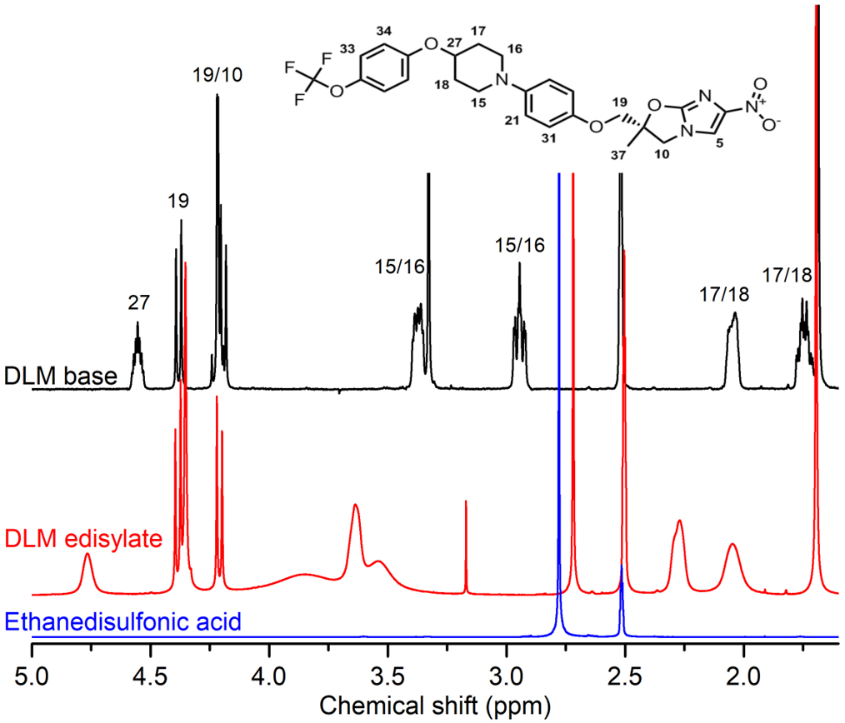


**Figure S4. Solution-state ^1^H NMR spectra of DLM free base and DLM edisylate in DMSO-d6.**


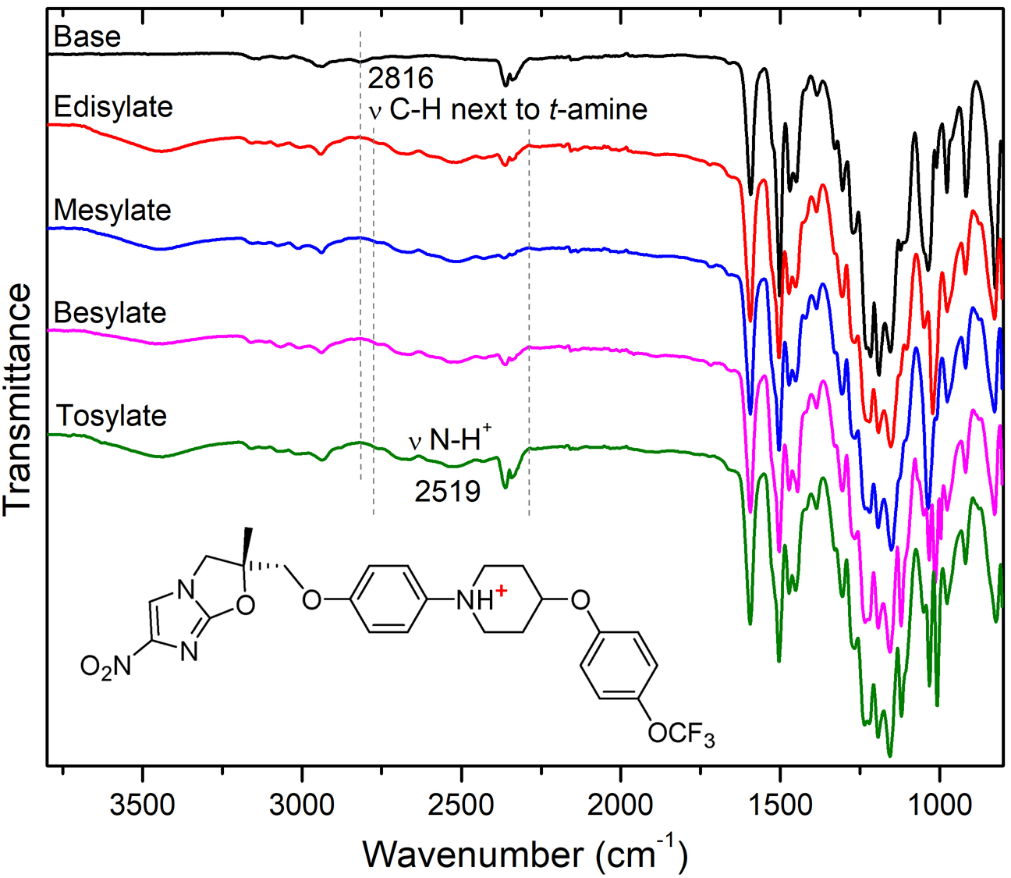


**Figure S5. FTIR spectra of amorphous DLM free base and sulfonate salts.**


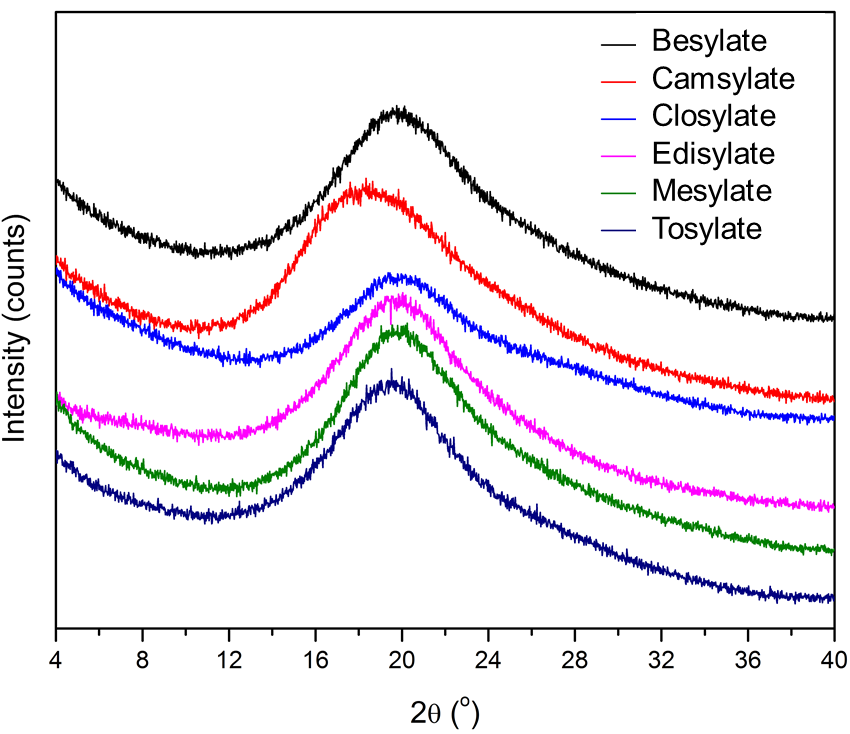


**Fig. S6. XRPD patterns of DLM sulfonate salts after 2 weeks storage at 40°C/75% RH open dish conditions.**


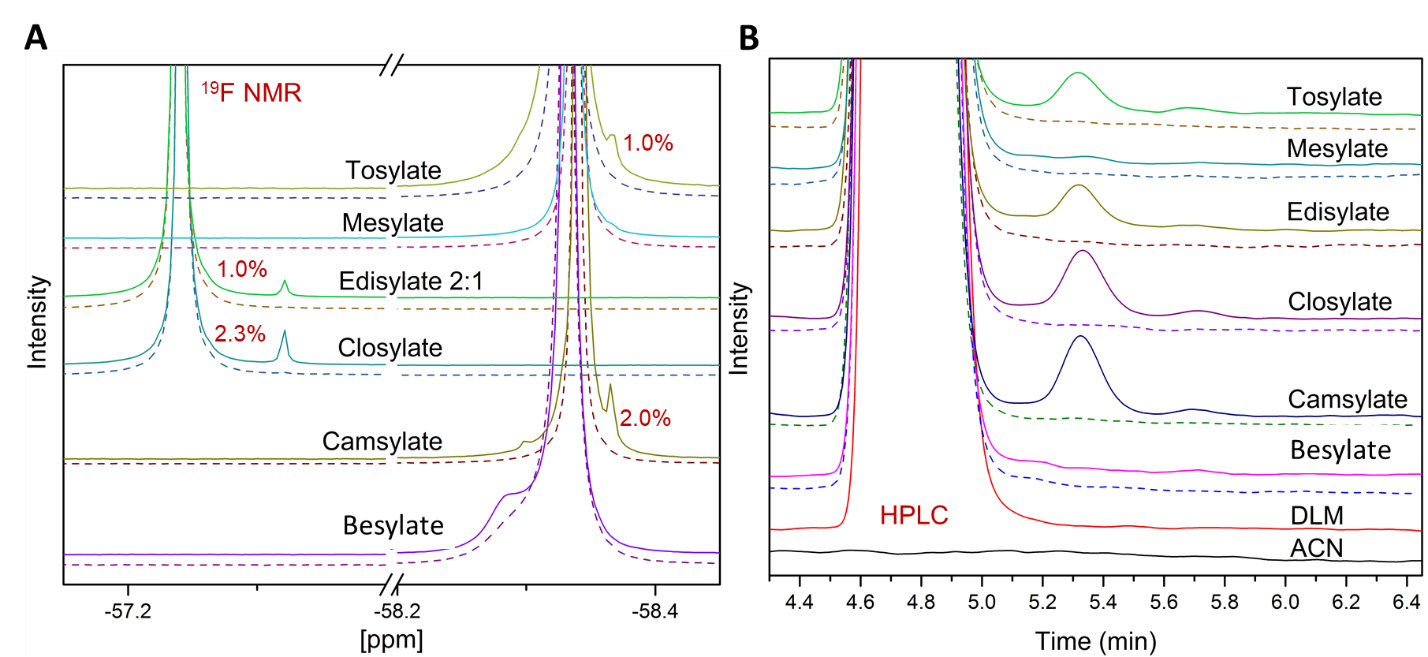


**Fig. S7. Chemical stability of DLM sulfonate salts after 2 weeks storage in accelerated condition characterized by (A) ^19^F-NMR spectroscopy and (B) HPLC. Dash lines indicate measurements of freshly prepared salts; solid lines depict results of samples after storage at 40°C/75% RH.**

*
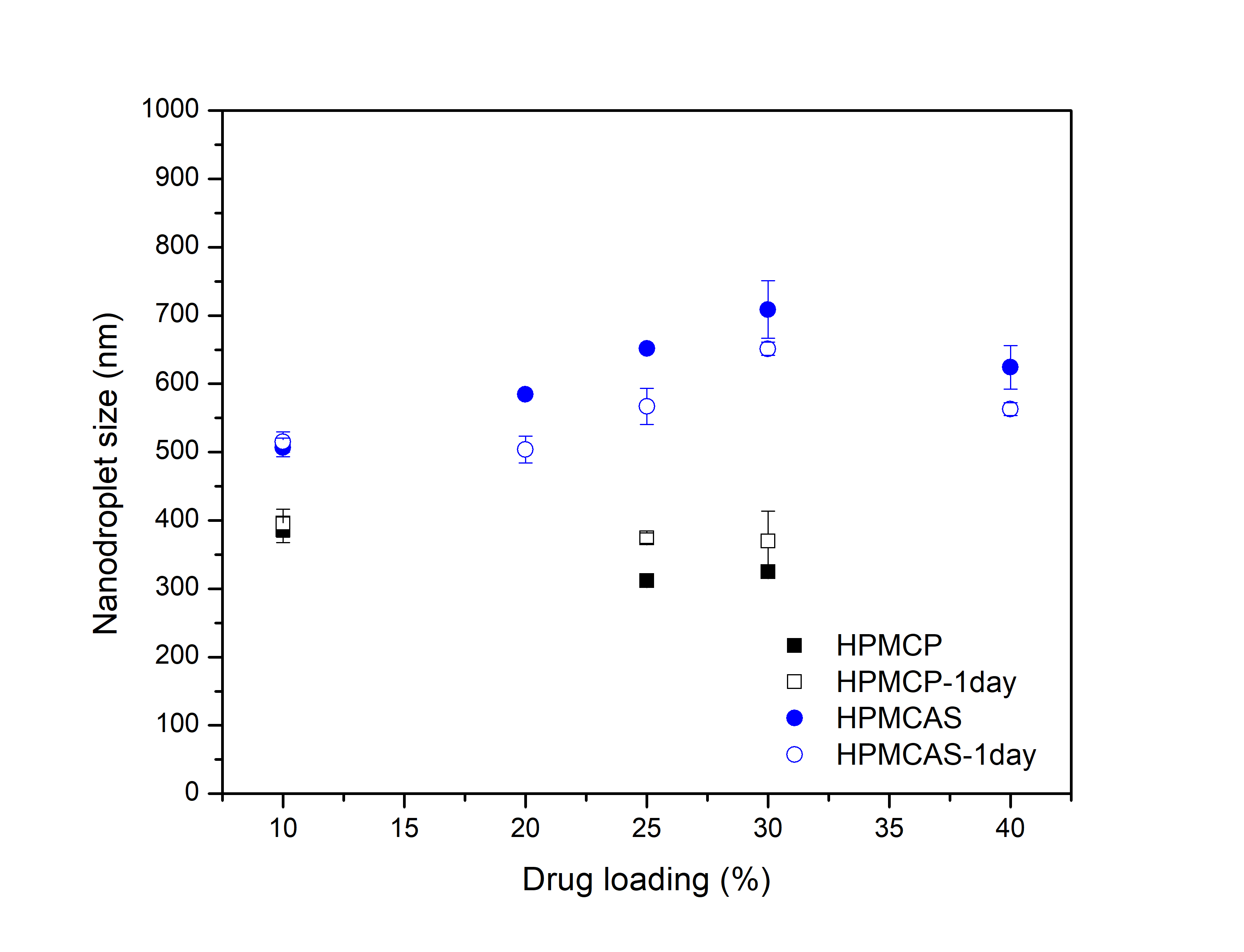
*

**Fig. S8. Size measurement of drug-rich nanodroplets generated from DLM-edisylate ASDs at different DLs immediately following ASD dissolution and 1-day post-dissolution in PBS pH 6.5.**

**pH 6.5.***
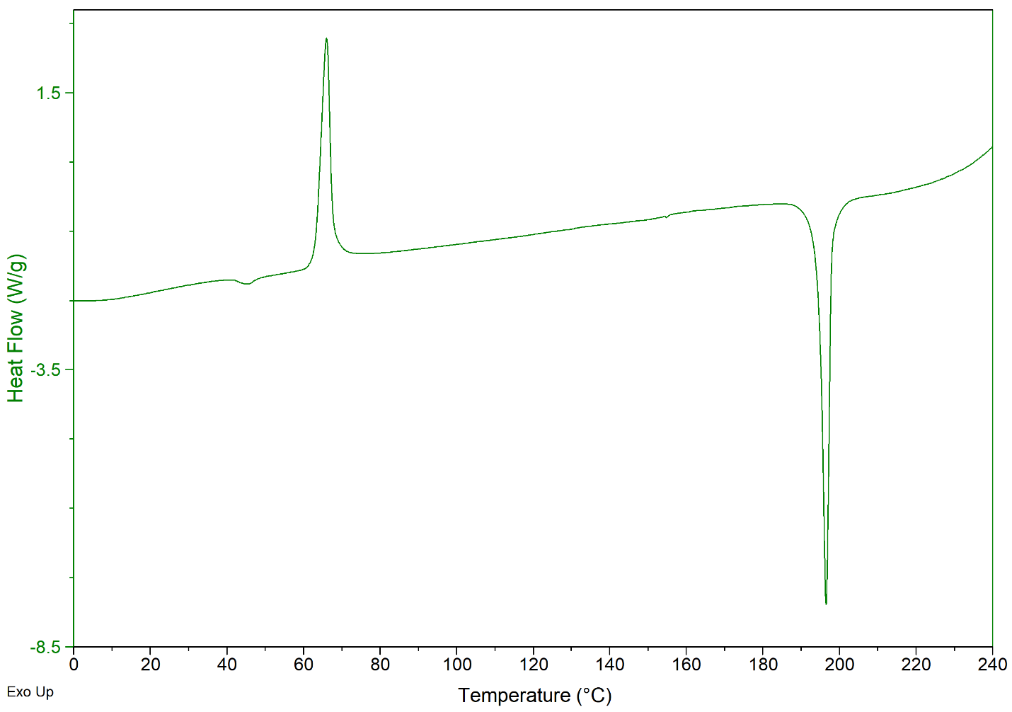
*

**Fig. S9. Differential scanning calorimetry thermograms showing fast recrystallization of initially glassy delamanid (obtained by quenching the melt in liquid nitrogen) upon heating to just above the glass transition temperature***.*

*
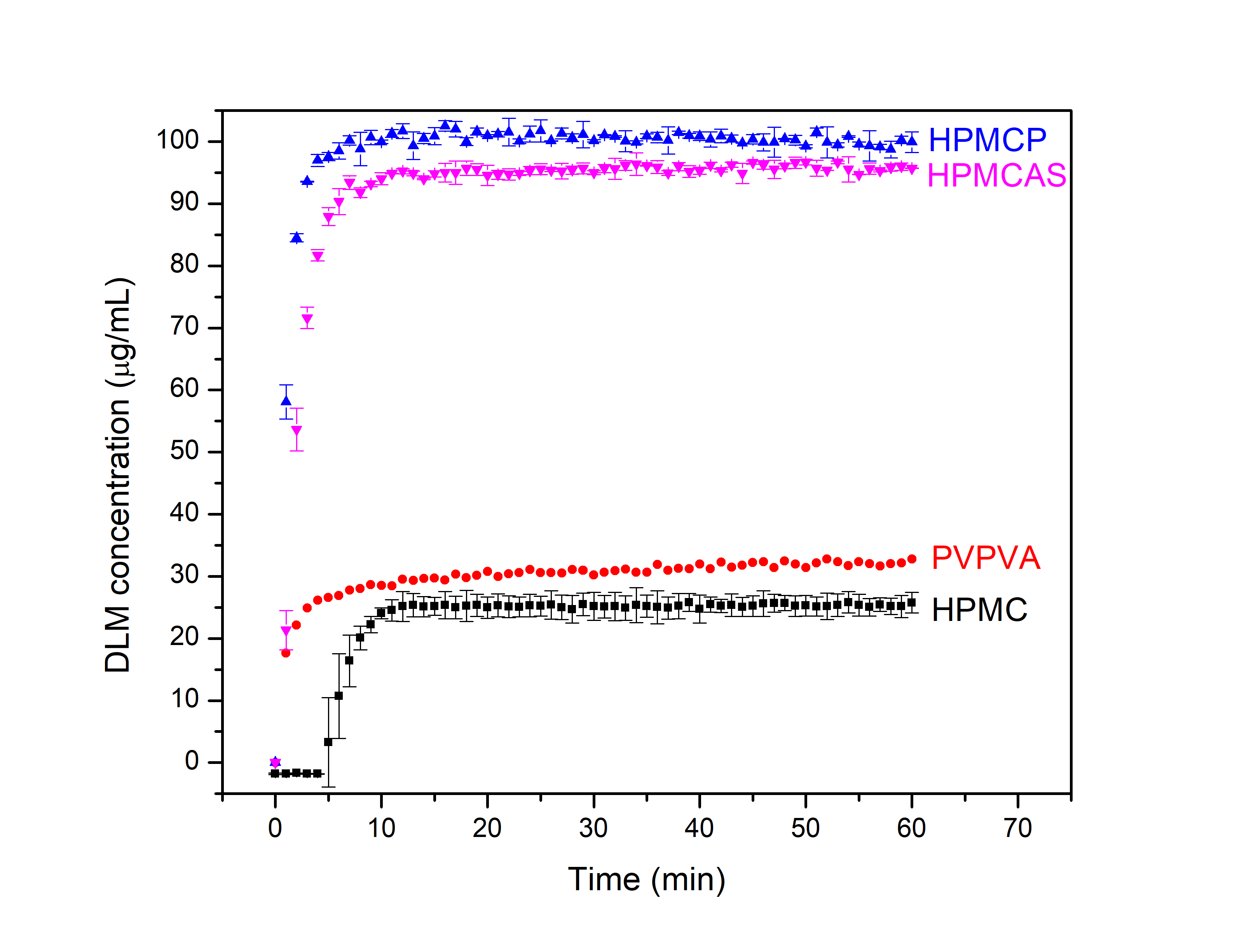
*

**Fig. S10. Dissolution profile of DLM-edisylate ASDs at 10% DL with different polymers.**

**References**

[1] L. Bighley, S. Berge, D. Monkhouse, Salt forms of drugs and absorption, Encyclopedia of Pharmaceutical Technology, 13 (1996) 453-499.

[2] J.P. Guthrie, Hydrolysis of esters of oxy acids: pKa values for strong acids; Brønsted relationship for attack of water at methyl; free energies of hydrolysis of esters of oxy acids; and a linear relationship between free energy of hydrolysis and pKa holding over a range of 20 pK units, Can. J. Chem., 56 (1978) 2342-2354.

[3] S.N. Black, E.A. Collier, R.J. Davey, R.J. Roberts, Structure, solubility, screening, and synthesis of molecular salts, J. Pharm. Sci., 96 (2007) 1053-1068.

[4] P.H. Stahl, C.G. Wermuth, Handbook of pharmaceutical salts: properties, selection and use, Chem. Int, 24 (2002) 21.

[5] H. Dong, H. Du, S.R. Wickramasinghe, X. Qian, The Effects of Chemical Substitution and Polymerization on the p K a Values of Sulfonic Acids, J. Phys. Chem. B, 113 (2009) 14094-14101.

[6] F. Cardellini, R. Germani, G. Cardinali, L. Corte, L. Roscini, N. Spreti, M. Tiecco, Room temperature deep eutectic solvents of (1 S)-(+)-10-camphorsulfonic acid and sulfobetaines: hydrogen bond-based mixtures with low ionicity and structure-dependent toxicity, RSC Adv., 5 (2015) 31772-31786.
